# Cardiac NF-κB Acetylation Increases While Nrf2-Related Gene Expression and Mitochondrial Activity Are Impaired during the Progression of Diabetes in UCD-T2DM Rats

**DOI:** 10.3390/antiox11050927

**Published:** 2022-05-09

**Authors:** Max A. Thorwald, Jose A. Godoy-Lugo, Ruben Rodriguez, Kimber L. Stanhope, James L. Graham, Peter J. Havel, Henry Jay Forman, Rudy M. Ortiz

**Affiliations:** 1 Leonard Davis School of Gerontology, University of Southern California, Los Angeles, CA 90017, USA; jgodoy4@ucmerced.edu (J.A.G.-L.); hforman@usc.edu (H.J.F.); 2 Department of Molecular and Cell Biology, School of Natural Sciences, University of California, Merced, CA 95343, USA; rortiz@ucmerced.edu; 3 Department of Cellular and Molecular Pharmacology, University of California, San Francisco, CA 94143, USA; ruben.rodriguez@ucsf.edu; 4 Department of Molecular Biosciences, School of Veterinary Medicine and Department of Nutrition, University of California, Davis, CA 95616, USA; klstanhope@ucdavis.edu (K.L.S.); jlgraham@ucdavis.edu (J.L.G.); pjhavel@ucdavis.edu (P.J.H.)

**Keywords:** mitochondrial dysfunction, diabetes, inflammation, antioxidants, oxidative stress

## Abstract

The onset of type II diabetes increases the heart’s susceptibility to oxidative damage because of the associated inflammation and diminished antioxidant response. Transcription factor NF-κB initiates inflammation while Nrf2 controls antioxidant defense. Current evidence suggests crosstalk between these transcription factors that may become dysregulated during type II diabetes mellitus (T2DM) manifestation. The objective of this study was to examine the dynamic changes that occur in both transcription factors and target genes during the progression of T2DM in the heart. Novel UC Davis T2DM (UCD-T2DM) rats at the following states were utilized: (1) lean, control Sprague-Dawley (SD; *n* = 7), (2) insulin-resistant pre-diabetic UCD-T2DM (Pre; *n* = 9), (3) 2-week recently diabetic UCD-T2DM (2Wk; *n* = 9), (4) 3-month diabetic UCD-T2DM (3Mo; *n* = 14), and (5) 6-month diabetic UCD-T2DM (6Mo; *n* = 9). NF-κB acetylation increased 2-fold in 3Mo and 6Mo diabetic animals compared to SD and Pre animals. Nox4 protein increased 4-fold by 6Mo compared to SD. Nrf2 translocation increased 82% in Pre compared to SD but fell 47% in 6Mo animals. GCLM protein fell 35% in 6Mo animals compared to Pre. Hmox1 mRNA decreased 45% in 6Mo animals compared to SD. These data suggest that during the progression of T2DM, NF-κB related genes increase while Nrf2 genes are suppressed or unchanged, perpetuating inflammation and a lesser ability to handle an oxidant burden altering the heart’s redox state. Collectively, these changes likely contribute to the diabetes-associated cardiovascular complications.

## 1. Introduction

Type II diabetes (T2DM) is a growing epidemic with more than 70% of its deaths caused by cardiovascular-related complications [1]. Insulin resistance and T2DM have been associated with chronic inflammation and impaired nucleophilic tone [2,3,4]. Nuclear factor kappa-light-chain-enhancer of activated B cells (NF-κB) is a transcription factor that primarily regulates inflammatory cascades by increasing cytokine levels to elicit immune responses, many of which directly contribute to increases in oxidant production [5]. Nuclear factor erythroid 2-related factor 2 (Nrf2) primarily regulates genes responsible for neutralizing excess oxidant production, xenobiotic detoxification, and NADPH replenishment [6]. While the mechanisms remain unclear, downstream targets of NF-κB and Nrf2 have been proposed to regulate each other. This regulation is problematic in diseases associated with NF-κB mediated chronic low-grade inflammation and subsequent oxidant generation such as T2DM [7,8].

NF-κB regulation occurs in the cytosol where it is bound to a family of proteins known as NF-κB inhibitors (IκB). IκBα is phosphorylated allowing NF-κB p65 to enter the nucleus where it transcribes a battery of genes associated with inflammation and apoptosis [5,9]. Tumor necrosis factor-alpha (TNFα), a potent inducer and product of NF-κB stimulation has been implicated in disrupting insulin’s action during periods of hyperglycemia [4]. Among other downstream targets associated with NF-κB gene expression are pro-oxidant enzymes NADPH oxidase 2 (Nox2) and NADPH oxidase 4 (Nox4), both present in cardiac tissue. Nox2 and 4 are both upregulated in the vasculature during the manifestation of T2DM and increase in response to TNFα stimulation [10,11,12]. Nox proteins produce either superoxide and/or hydrogen peroxide, and their overexpression has been well documented in inducing cardiac hypertrophy and oxidative damage. 

Transcription factor Nrf2 is sequestered in the cytosol by kelch-like ECH-associated protein 1 (Keap1) [13] and is liberated after modification of Keap1′s cysteines. Nrf2 translocates to the nucleus binding the electrophile response element (EpRE), initiating gene expression [14,15]. Nrf2 increases antioxidant expression in response to a rise in oxidant production; however, during T2DM nuclear Nrf2 translocation and/or antioxidant induction is impaired. Cellular damage accrues each time oxidant levels increase. Degradation of Nrf2 is partially mediated by nuclear glycogen synthase kinase-3 Beta (GSK3β) through β-TrCP [16,17,18]. Thus, nuclear Nrf2 import may still be dampened impairing phase II gene responses.

Mitochondria are the largest producers of oxidants [19,20,21] generating hydrogen peroxide (H_2_O_2_) and peroxynitrite (ONOO^−^) even under unstressed conditions. H_2_O_2_ is derived from superoxide (O_2_^·−^) while ONOO^−^ is derived from the reaction of nitric oxide (NO) with O_2_^·−^. During T2DM, oxidant production increases from mitochondrial dysfunction [22]. Cardiac mitochondria contain numerous antioxidants including proteins belonging to superoxide dismutase [23], catalase [24], glutathione peroxidase, and transferase families [25]. The latter utilize glutathione (GSH) to mitigate excess oxidant production and detoxify lipid peroxidation products [25]. GSH is produced in the cytosol and imported into the mitochondria for these purposes. However, genes responsible for GSH biosynthesis or utilization are under Nrf2′s control and nuclear Nrf2 protein is low during T2DM likely from impaired Nrf2 translocation and/or enhanced degradation. Increases in mitochondrial oxidant production and decreases in antioxidant defense cause damage to accrue increasing mitophagy through localization of parkin to the mitochondria [19,26,27]. 

NF-κB and Nrf2 have been studied extensively in numerous diseases including T2DM often with evidence of their crosstalk [7,28,29,30,31,32]. While their crosstalk remains unclear, it is proposed that upon nuclear entry, NF-κB and Nrf2 compete for CREB which is necessary for either to bind their target sequence of DNA [33]. Conversely, some argue that NF-κB is required for Nrf2 nuclear import likely through a signaling event caused by prior activation of NF-κB [34]. Because these transcription factors are often measured independently, we aimed to track their status as T2DM progresses and in a more physiologically relevant but underutilized model of T2DM [35,36]. Here, we show that NF-κB activation and subsequent target gene expression increases while Nrf2 becomes unresponsive in late T2DM. 

## 2. Methods

All experimental procedures were reviewed and approved by the institutional animal care and use committee of the University of California, Davis (08/07/2008–08/07/2011 IACUC #: 15011).

### 2.1. Animals

UCD-T2DM rats were generated by crossing obese Sprague-Dawley rats with adult-onset obesity and insulin resistance with ZDF lean rats that have a defect in β-cell/islet function, but were homozygous, wild-type for the leptin receptor [35,37]. The UCD-T2DM rat has been characterized and validated in more than 20 subsequent peer-reviewed publications [36,37,38] and closely resembles the pathophysiology of T2DM in humans. T2DM progression was determined by weekly intravenous glucose tolerance as previously described [35]. T2DM manifestation was characterized as having >200mg/dL nonfasting blood glucose for 2 consecutive weeks. Animals in the pre-diabetic group were age matched but did not meet the >200mg/dL criteria for T2DM. Pre animals had elevated fasting plasma insulin compared to SD [35]. Lean Sprague-Dawley (SD; *n* = 7), pre-diabetic UCD-T2DM (Pre; *n* = 9), 2-week diabetic UCD-T2DM (2Wk; *n* = 9), and 3-month diabetic UCD-T2DM (3Mo; *n* = 14) were all 5.5 months of age at the time of tissue collection. The 6-month diabetic UCD-T2DM (6Mo; *n* = 9) rats were approximately 90 days older than all other rats as previously described [37]. Phenotypic data for the animals used in this study has been previously described [35,37] (Table 1). All animals were maintained in a specific pathogen-free facility with a 14 h light, 10 h dark cycle. All animals were given *ad libitum* access to standard laboratory rat chow (Harlan Laboratories, Indianapolis, IN) and water. Prior to dissection, animals were fasted for 13 h and blood was collected via tail vein into EDTA-coated tubes which was used for subsequent assays. Animals were given a 200 mg/kg IP injection of pentobarbital sodium and exsanguinated via cardiac puncture. Heart tissue was snap frozen in liquid nitrogen and stored at −80 °C [33]. 

### 2.2. Western Blot Analyses

A 20 mg piece of frozen mixed ventricle was homogenized in 125 μL of sucrose buffer for subsequent extraction of the mitochondrial, nuclear, and cytosolic fractions [39]. Proteins were assayed as previously described [40] and incubated for 16 h with primary antibodies (diluted 1:100 to 1:4000) against GCLC, GCLM (provided by Dr. Forman), Nox4, Vdac1 (Abcam, Cambridge, MA, USA), NF-κB p65, acetylated-NF-κB (Lys 310), IκBα, p-IκBα, H3, (Cell Signaling, Danvers, MA, USA), Keap1, Nrf2, GSK3β, Parkin, GAPDH (Santa Cruz Biotechnology, Santa Cruz, CA, USA), 4-hydroxy-2-nonenal (4HNE), Nox2 (EMD Millipore, Burlington, MA, USA), and Hmox1 (Proteintech, Rosemont, IL, USA). Blots were visualized using an Odyssey system (LI-COR Biosciences) and quantified using ImageJ. Nuclear and cytosolic extractions were tested for purity against H3 and GAPDH [39]. Mitochondrial extractions were also tested for purity with Vdac1 and GAPDH. In addition to consistently loading the same amount of total protein per well, values were further normalized by correcting with the densitometry values of Ponceau S staining [41]. Three Western blots were run per protein to include all available biological replicates. All blots contained no less than 2 representative samples per group. 

### 2.3. Real-Time Quantitative PCR Analyses

Total RNA was isolated using TRIzol reagent (Invitrogen, Carlsbad, CA, USA). Genomic DNA was degraded using DNase I enzyme (Roche, Indianapolis, IN, USA). Total RNA purity was confirmed by absorbance at 260/280 and integrity by 1% agarose gel electrophoresis. Complementary DNA was reverse transcribed from genomic DNA-free RNA (2 μg) using the High-Capacity cDNA Reverse Transcription Kit (Applied Biosystems, Foster City, CA, USA) and oligio-dT. Quantitative PCR reactions were performed in duplicate using an equivalent to 100 ng of RNA and specific primers for Keap1, GSK3β, Nrf2, NF-κB, IκBα, Bach1, GCLM, GCLC, Hmox1, and Nox4 (Table 2). Gene expression was normalized using B2M expression and values were analyzed using the comparative double delta-CT method. 

### 2.4. Biochemical Analyses

Complex I, complex II (Abcam, Cambridge, MA, USA), and TNFα (Meso Scale Discovery, Rockville, MD, USA) assays were measured by ELISA. All samples were analyzed in duplicate and run in a single assay with intra-assay and percent coefficients of variability of less than 10% for all assays.

### 2.5. Statistics

Means (±SEM) were compared by ANOVA. Means were considered significantly different at *p* < 0.05 using Tukey’s HSD. Outliers were removed and replaced with the group mean [42]. Statistical analyses were performed with GraphPad Prism 8 software (Graphpad, San Diego, CA, USA). 

## 3. Results

### 3.1. Body Mass, Glucose, and Insulin Measurements 

Phenotype data was collected to track the progression of T2DM. Body mass was increased in Pre (56%) and 2Wk (60%) animals compared to SD. Body mass was 26% lower in 3Mo remained lower in 6Mo animals compared to SD. Fasting blood glucose increased 16% in Pre and 2Wk animals compared to SD. Fasting blood glucose further increased 129% in 3Mo and 3.5-fold in 6Mo old animals, respectively. Non-fasting blood glucose was 30% higher in Pre animals compared to SD. Non-fasting blood glucose further increased in 2Wk (1.5-fold), 3Mo (3.5-fold), and 6Mo (4-fold) animals compared to SD. Glycated hemoglobin (HbA1c) increased 4-fold in 2Wk animals compared to SD. HbA1c levels further increased 3-fold in 3Mo and 6Mo animals. Insulin levels were 2-fold higher in pre-diabetic and 3-fold in 2-week diabetic animals. Insulin levels were reduced 63% in 3Mo and 80% in 6Mo animals compared to 2Wk animals (Table 1).

### 3.2. Cardiac NF-κB Signaling Is Increased in UCD-T2DM Rats as Diabetes Progresses

**Figure 1:**
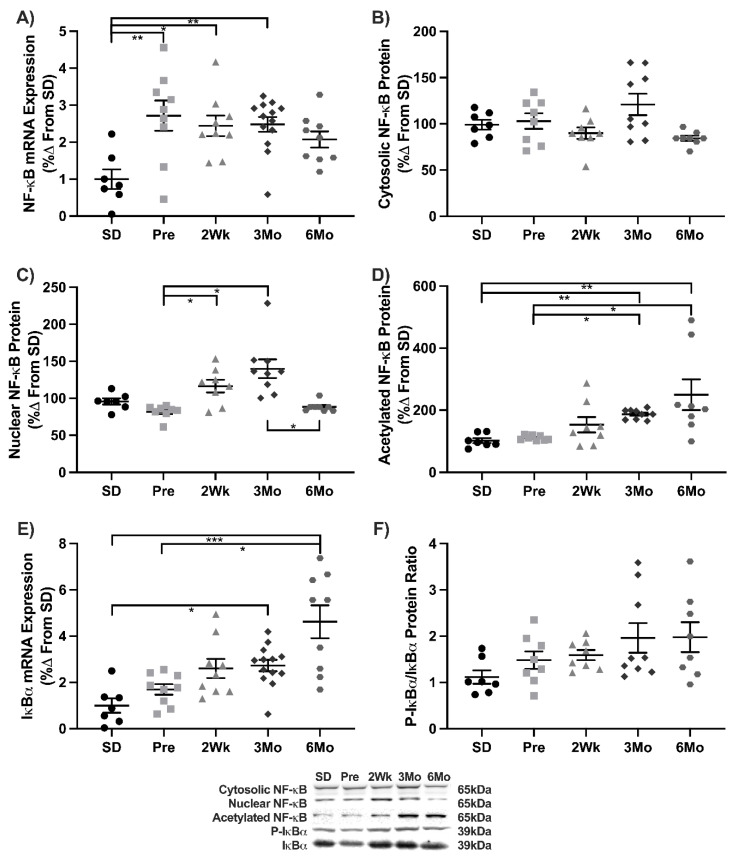
NF-κB signaling during the progression of T2DM. Mean ± SEM values of (**A**) NF-κB mRNA, (**B**) cytosolic NF-κB protein, (**C**) nuclear NF-κB protein, (**D**) acetylated Lys310 nuclear NF-κB protein, (**E**), IκBα mRNA, and (**F**) the ratio of phosphorylated to native IκBα protein in Sprague-Dawley (SD; *n* = 7), pre-diabetic UCD-T2DM (Pre; *n* = 9), 2-week diabetic UCD (2Wk; *n* = 9), 3-month diabetic UCD-T2DM (3Mo; *n* = 13), and 6-month diabetic UCD-T2DM (6Mo; *n* = 9) rats. * *p* < 0.05, ** *p* < 0.01, *** *p* < 0.001.

Chronic inflammation is synonymous with T2DM. Canonical components of NF-κB signaling were measured to determine if similar outcomes occurred in UCD-T2DM rats. NF-κB transcripts increased 2.5-fold in Pre compared to SD and remained elevated in 2Wk and 3Mo groups (Figure 1A). No changes were observed in cytosolic NF-κB expression (Figure 1B). Nuclear NF-κB increased in 2Wk (43%) and 3Mo (71%) animals compared to Pre. Nuclear NF-κB decreased 37% in 6Mo animals compared to 3Mo (Figure 1C). No differences were observed in NF-κB translocation (not shown). Acetylation of Lys310 on Nuclear NF-κB protein increased 2-fold in 3Mo and 6Mo animals compared to SD and Pre (Figure 1D). IκBα transcripts increased in 2- and 3-fold in 3Mo and 6Mo animals compared to SD, respectively. 6Mo animals also had 2-fold more IκBα transcripts than Pre (Figure 1E). No changes were observed in the ratio of phosphorylated and native IκBα, protein (Figure 1F). Overall, these data suggest that as diabetes progresses, cardiac NF-κB activation increases.

### 3.3. NF-κB Associated Inflammatory Genes Increase as T2DM Progresses 

**Figure 2:**
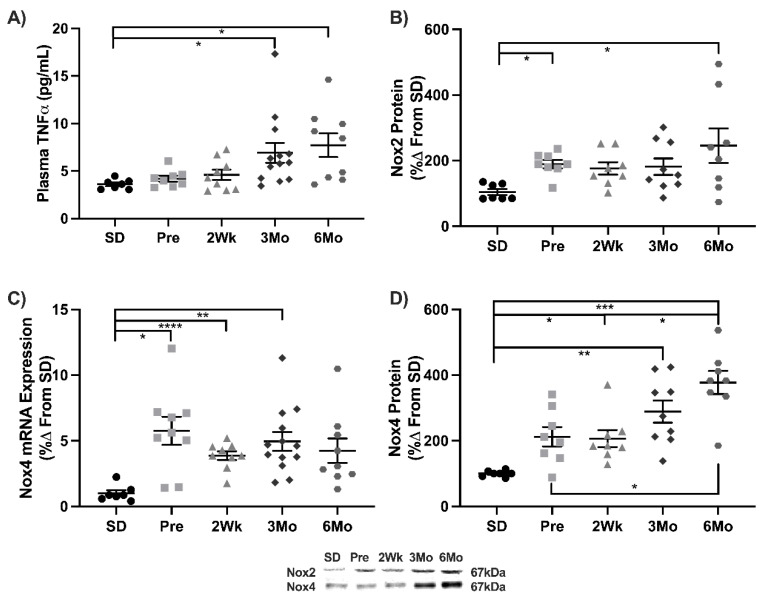
Downstream targets of NF-κB activation. Mean ± SEM values of (**A**) plasma TNFα, (**B**) Nox2 protein, (**C**) Nox4 mRNA, and (**D**) Nox4 protein in Sprague-Dawley (SD; *n* = 7), pre-diabetic UCD-T2DM (Pre; *n* = 9), 2-week diabetic UCD (2Wk; *n* = 9), 3-month diabetic UCD-T2DM (3Mo; *n* = 13), and 6-month diabetic UCD-T2DM (6Mo; *n* = 9) rats. * *p* < 0.05, ** *p* < 0.01, *** *p* < 0.001, **** *p* < 0.0001.

Several downstream targets responsible for heightened inflammation and oxidant production were measured to determine the increase in NF-κB activation altered gene expression. Plasma TNFα levels increased 112% in 3Mo and 134% in 6Mo animals compared to SD (Figure 2A). Nox2 protein increased 82% in Pre animals compared to SD and increased further by 134% in 6Mo animals (Figure 2B). Nox4 transcripts increased 4-fold in Pre, 2Wk, and 3Mo animals compared to SD (Figure 2C). Nox4 expression increased 2-, 3-, and 4-fold in 2Wk, 3Mo, and 6Mo old animals compared to SD, respectively. Nox4 expression was increased 77% and 83% in 6Mo animals compared to Pre and 2Wk, respectively (Figure 2D). 

### 3.4. Nrf2 Signaling Is Impaired or Unresponsive in UCD-T2DM Hearts

**Figure 3:**
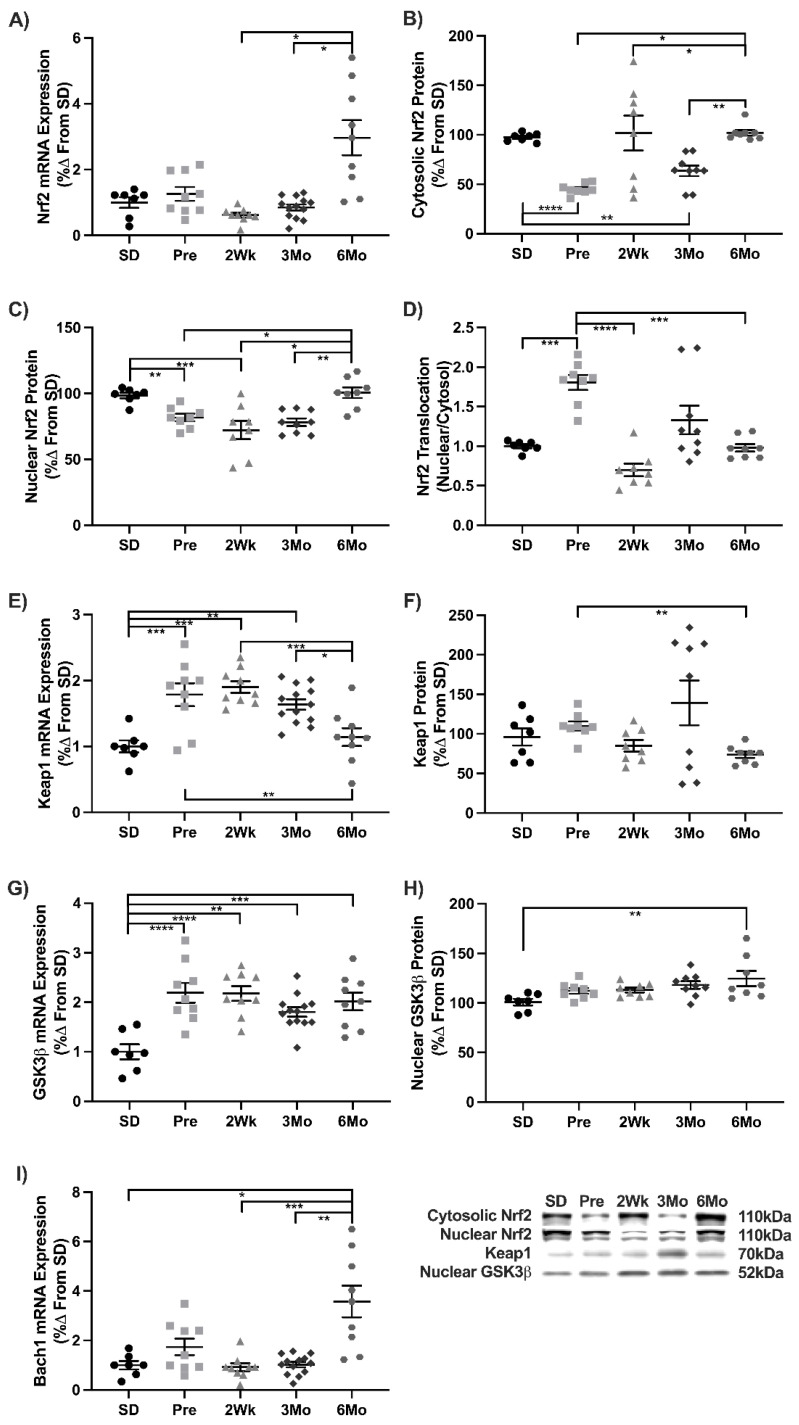
Nrf2 signaling during the progression of T2DM. Mean ± SEM values of (**A**) Nrf2 mRNA, (**B**) cytosolic Nrf2 protein, (**C**) nuclear Nrf2 protein, (**D**) Nrf2 translocation, (**E**) Keap1 mRNA, (**F**) Keap1 protein, (**G**) GSK3β mRNA, (**H**) nuclear GSK3β protein, and (**I**) Bach1 mRNA in Sprague-Dawley (SD; *n* = 7), pre-diabetic UCD-T2DM (Pre; *n* = 9), 2-week diabetic UCD (2Wk; *n* = 9), 3-month diabetic UCD-T2DM (3Mo; *n* = 13), and 6-month diabetic UCD-T2DM (6Mo; *n* = 9) rats. * *p* < 0.05, ** *p* < 0.01, *** *p* < 0.001, **** *p* < 0.0001.

Nrf2 is the master regulator of antioxidant gene production and was measured to ascertain its status during T2DM progression. Nrf2 transcripts were unchanged except in 6Mo animals where they were 2- and 4-fold higher than 2Wk and 3Mo animals, respectively (Figure 3A). Cytosolic Nrf2 decreased 54% in Pre and 33% 2Wk animals compared to SD, respectively. Cytosolic Nrf2 levels returned to baseline at 6Mo (Figure 3B). Nuclear Nrf2 was decreased 15% in Pre and 25% in 2Wk animals compared to SD. The 6Mo animals returned to baseline (Figure 3C). Nrf2 translocation increased 82% in Pre compared to SD. Nrf2 translocation was decreased in 2Wk (−60%) and 6Mo (−47%) animals compared to Pre (Figure 3D). Keap1 transcripts increased 78% in Pre compared to SD and remained elevated in 2Wk (+90%) and 3Mo (+63%) groups. Transcripts in 6Mo animals returned to baseline (Figure 3E). Keap1 expression decreased 33% in 6Mo animals compared to Pre (Figure 3F). GSK3β transcripts increased 2-fold in Pre animals compared to SD and remained elevated in 2Wk, 3MO, and 6Mo old animals (Figure 3G). Nuclear GSK3β expression increased by 23% in 6Mo animals compared to SD (Figure 3H). Bach1 transcripts were unchanged except in the 6Mo animals where they increased 3.5-fold compared to the SD, 2Wk, and 3Mo groups (Figure 3I). These results demonstrate that Nrf2 signaling is impacted negatively during the progression of T2DM. 

### 3.5. Nrf2 Related Genes Are Unaltered or Reduced as T2DM Progresses

**Figure 4:**
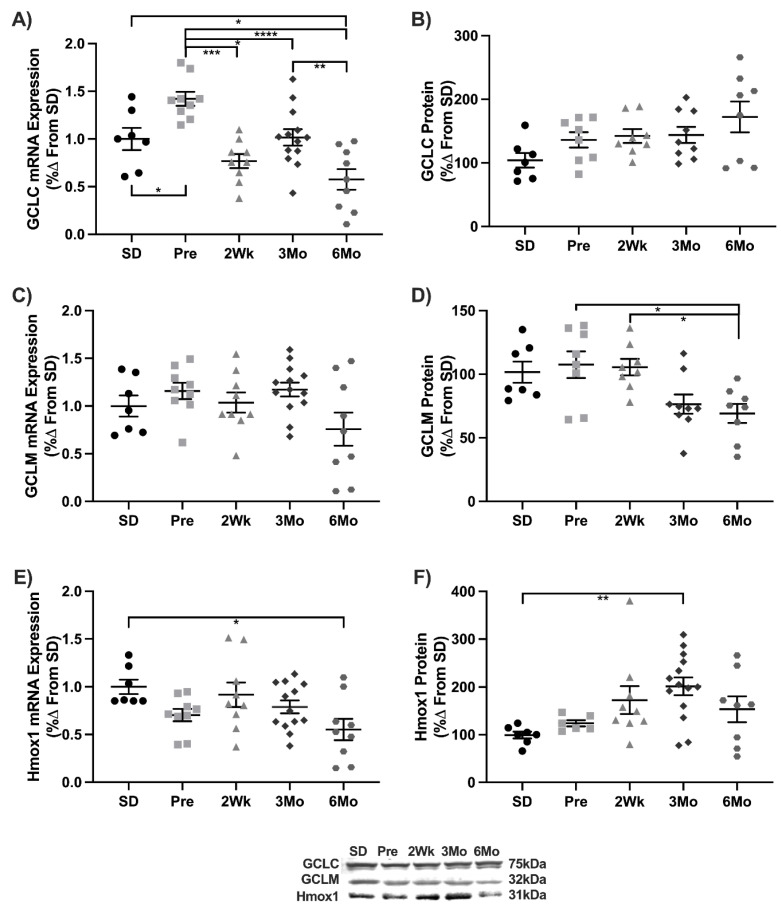
Downstream targets of cardiac Nrf2 activation are decreased or unchanged six months after the onset of T2DM. Mean ± SEM values of (**A**) GCLC mRNA, (**B**) GCLC protein, (**C**) GCLM mRNA, (**D**) GCLM protein, (**E**) Hmox1 mRNA, and (**F**) Hmox1 protein in Sprague-Dawley (SD; *n* = 7), pre-diabetic UCD-T2DM (Pre; *n* = 9), 2-week diabetic UCD (2Wk; *n* = 9), 3-month diabetic UCD-T2DM (3Mo; *n* = 14), and 6-month diabetic UCD-T2DM (6Mo; *n* = 9) rats. * *p* < 0.05, ** *p* < 0.01, *** *p* < 0.001, **** *p* < 0.0001.

GCLC transcripts increased in Pre animals 42% compared to SD. GCLC transcripts decreased in 2Wk (−46%), 3Mo (−30), and 6Mo (−60%) animals compared to Pre. The 6Mo animals had the lowest number of transcripts with a 43% reduction compared to SD and 3Mo animals (Figure 4A). No changes were observed in GCLC protein (Figure 4B) or GCLM transcripts (Figure 4C). GCLM expression was decreased 35% in 6Mo animals compared to Pre and 2Wk animals (Figure 4D). Hmox1 transcripts decreased 45% in 6Mo animals compared to SD (Figure 4E). Hmox1 protein increased 67% in 3Mo (Figure 4F). These results suggest that reductions in Nrf2-related genes in concert with heightened NF-κB genes may make the heart more susceptible to oxidative damage.

### 3.6. UCD-T2DM Rats Have Mitochondrial Dysfunction and Increased Mitochondrial Damage Consistent with T2DM Pathology 

**Figure 5:**
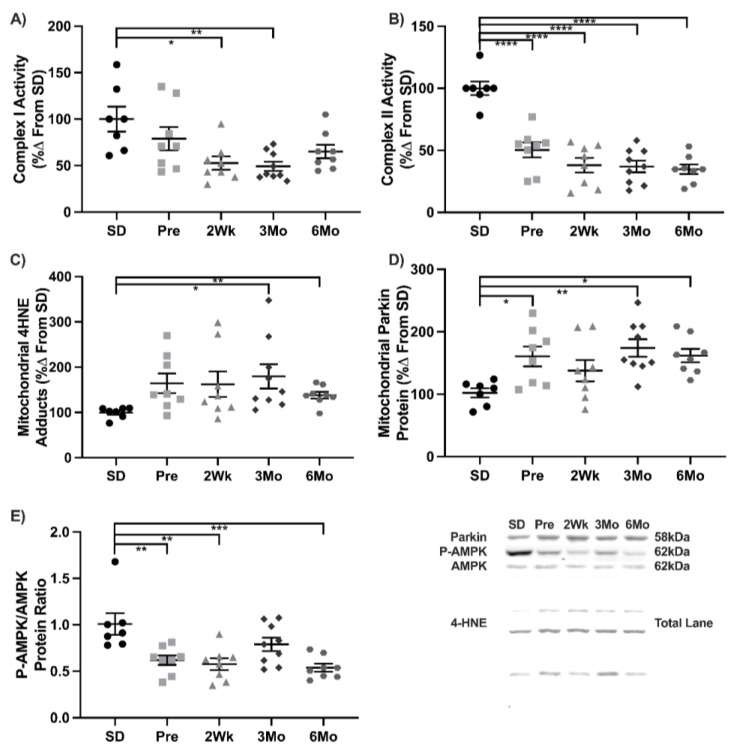
Mitochondria dysfunction and oxidative damage increase as T2DM progresses. Mean ± SEM values of (**A**) Complex I activity, (**B**) Complex II activity, (**C**) mitochondrial 4HNE adducts, (**D**) mitochondrial Parkin protein, and (**E**) ratio of phosphorylated (T172) to native AMPK protein in Sprague-Dawley (SD; *n* = 7), pre-diabetic UCD-T2DM (Pre; *n* = 9), 2-week diabetic UCD (2Wk; *n* = 9), 3-month diabetic UCD-T2DM (3Mo; *n* = 13), and 6-month diabetic UCD-T2DM (6Mo; *n* = 9) rats. * *p* < 0.05, ** *p* < 0.01, *** *p* < 0.001, **** *p* < 0.0001.

Mitochondrial function and damage were measured to determine if the mitochondrial dysfunction present in T2DM and other animals was observed in UCD-T2DM rats. Mitochondrial complex I activity decreased 47% in the 2Wk old ventricle compared to SD and remained suppressed in 3Mo animals by 51% (Figure 5A). Complex II was lowered in the Pre animals 50% compared to SD. Development of T2DM further decreased complex II activity in 2Wk old animals compared to SD at 62% and remained suppressed in 3Mo (−64%) and 6Mo (−67%) animals (Figure 5B). Mitochondrial HNE content was measured to determine the oxidative damage accrued in the mitochondria. HNE levels increased at 73% in 3Mo and remained elevated in 6Mo (+37%) old animals (Figure 5C). Mitochondrial parkin increased in the mitochondrial fraction 60% in the Pre animals compared to SD. This increase was sustained in the 3Mo (+74%) and 6Mo (+56%) animals (Figure 5D). The ratio of native and Phosphorylated AMPK (Thr172) demonstrated a reduction in Pre animals by 32%. These reductions were observed in 2Wk (−43%) and 6Mo (−47%) animals as well (Figure 5E). Collectively, these decreases in mitochondrial complex activity and increases in HNE and parkin suggest cardiac mitochondrial dysfunction in the UCD-T2DM rats consistent with T2DM pathogenesis. 

## 4. Discussion 

Chronic low-grade inflammation during T2DM and obesity are established characteristics due to activation of NF-κB. While the stimulation of inflammatory cascades and suppression of antioxidants systems are well documented in T2DM, here we provide data demonstrating the temporal enhancement of cardiac NF-κB associated with increases in acetylation and Nox proteins, and diminished activation of Nrf2 with the transition from insulin resistance to T2DM. 

NF-κB is tethered in the cytosol by IκBα, which requires phosphorylation to liberate NF-κB and allow for its nuclear import. Despite an increase in IκBα mRNA during the progression of T2DM, this increase did not translate to a detectable increase in IκBα phosphorylation. NF-κB mRNA increased in the prediabetic animals and remained elevated but did not result in an increase in cytosolic NF-κB protein suggesting that either the increase in mRNA expression was not sufficient to maintain elevated protein levels or NF-κB turnover increased as T2DM progressed. However, nuclear NF-κB p65 increased as T2DM progressed except in the 6-month diabetic hearts where NF-κB levels were the same as the control animals suggesting that the lack of a decrease in the cytosol was a factor of nuclear accumulation. Despite the lack of nuclear NF-κB in the insulin-resistant and late-stage diabetic animals, acetylation of nuclear NF-κB at Lys310 rose sharply with the progression of T2DM. This suggests acetylation of NF-κB may be more relevant for assessing NF-κB activation rather than nuclear NF-κB protein. While acetylated lysine 310 bears no consequence on DNA binding, it is essential for full transcriptional activity of NF-κB p65 illustrating increased activation during disease progression [43,44]. 

Downstream targets of NF-κB were measured to determine if increased p65 acetylation translated into increased target genes. Plasma TNFα levels matched changes in NF-κB activation. TNFα is a potent inducer of oxidant production through the Nox family, which directly contributes to oxidant generation through use of NAPDH [5,11,45]. Nox2 and Nox4 are the predominantly expressed isoforms in the cardiovascular system with Nox4 regulated primarily by mRNA copy number [46]. Nox expression increased in the insulin-resistant animals preceding nuclear NF-κB increases suggesting their assemblage may occur before the onset of chronic inflammation. Increases in the Nox family during insulin resistance has been largely attributed to the renin–angiotensin system stimulation in other diabetic models [47]. NF-κB acetylation paralleled the increase in plasma TNFα levels and Nox proteins in 3- and 6-month hearts suggesting that acetylation and TNFα may both enhance Nox expression in late T2DM. These increases are characteristic of the chronic, systemic inflammation associated with oxidant generation during the manifestation of T2DM [48]. 

Meanwhile Nrf2, which is responsible for initiating transcription of genes involved in mitigating excess oxidant production NAPDH, decreased or was unchanged during T2DM progression. Nuclear Nrf2 accumulation does not occur without modification of inhibitory cytosolic protein Keap1. Keap1 transcripts increased in the insulin-resistant hearts and remained elevated until 6-month diabetic state. Keap1 protein matched mRNA and was also lowered at 6 months. Nrf2 translocation increased in the insulin resistant animals but decreased in the 2-week and 6-month diabetic hearts suggesting that Nrf2 is functional during insulin resistance but becomes dysregulated shortly after the onset of T2DM. While the levels of Nrf2 mRNA, and cytosolic and nuclear protein content increased in the 6-month diabetic hearts, nuclear Nrf2 inhibition may impair activation during late T2DM. GSK3β has been proposed to facilitate degradation of nuclear Nrf2 [16]. Nuclear GSK3β increased in late-stage T2DM potentially stifling Nrf2 activation. Bach1, a known competitor of EpRE binding [49,50], was unchanged during the diabetes progression; however, in 6-month diabetic hearts, Bach1 transcripts increased suggesting that nuclear Nrf2′s potential to bind to the EpRE and initiate transcription of target genes during advanced diabetes may be inhibited by competition of Bach1. Collectively, these data demonstrate that despite the increase in Nrf2 mRNA and relatively unaltered protein levels by 6 months, increases in nuclear regulators GSK3β and Bach1 likely limit Nrf2 activation. 

To determine if the absence of Nrf2 translocation impacted its target genes, we measured two critical enzymes for the GSH cycle. GSH is the most abundant, non-enzymatic antioxidant whose production is altered in diabetic hearts [51]. During cellular stress, GSH increases to counter the increases in oxidants. Glutamate cysteine ligase catalytic subunit (GCLC) and glutamate cysteine ligase modifier subunit (GCLM) dimerize to produce glutathione cysteine ligase (GCL). GCLC catalyzes the production of gamma-glutamylcysteine while GCLM increases the activity of GCLC greatly [52] and therefore is the rate limiting subunit for GSH synthesis. GCLC increased in the insulin resistant hearts but decreased following the onset of diabetes. No changes were observed in GCLC protein; however, GCLM protein decreased in the 6-month hearts. GCLM reductions suggest GSH biosynthesis is impaired 6 months after the onset of diabetes. Heme oxygenase 1 (Hmox1), another Nrf2 target gene involved in heme metabolism produces anti-inflammatory byproduct carbon monoxide (CO) which is known to negatively regulate NF-κB target genes [7,53]. Hmox1 mRNA was decreased; however, protein levels were increased by 3 months but were not different at 6 months, consistent with our other findings. Downregulation of Hmox1 mRNA and lack of response in Hmox1 protein at 6 months provides further evidence of the impaired Nrf2 activity. Blunted Nrf2 translocation further suggests that heightened NF-κB-mediated inflammation may impair Nrf2 activity and subsequent antioxidant defense during the progression of T2DM.

Energy balance is essential to antioxidant function where NADPH is required for many redox reactions [54]. Mitochondrial dysfunction is a hallmark of T2DM and was measured to assess the severity of the mitochondrial dysfunction in relation to the simultaneous changes in NF-κB and Nrf2 associated with the progression of T2DM [22]. Complex I and complex II decreased as diabetes progressed suggesting that the mitochondrial electron transport is impaired during the progression of T2DM. Dysfunction in the electron transport chain has been linked to increased oxidant production [55]. Parkin accumulates on the outside of damaged mitochondria inducing mitophagy [26,27]. The early and sustained increase in parkin suggests that the progression of T2DM was associated damaged mitochondria increasing the potential for mitophagy and altered mitochondrial function. Additionally, mitochondrial 4-HNE adducts, a consequence of lipid peroxidation, increased in late stage T2DM animals providing further evidence of the increase in mitochondrial damage. 4-HNE during the initiation of insulin resistance is consistent with other T2DM models [40]. AMPK acts as an energy sensor where phosphorylation of AMPK increases uptake of glucose and fatty acids. In as early as the pre-diabetic state, the ratio of phosphorylated to native AMPK decreased suggesting that cellular energetics were disrupted as a likely consequence of insulin resistance. These data suggest that cardiac mitochondria progressively worsen with the development of T2DM, paralleling the increased acetylation of NF-κB and impairment in Nrf2. Reductions in energy production and Nrf2-related antioxidants would increase the susceptibility of oxidative damage in the diabetic heart. 

These findings simultaneously track temporal alterations in two key transcription factors involved in T2DM and highlight that increased NF-κB and blunted Nrf2-mediated antioxidant responses are key contributors to T2DM. Furthermore, increased acetylation of NF-κB was associated with increased plasma TNFα and Nox protein expression, likely contributing to an increase in oxidant production in the diabetic heart while antioxidant defense and mitochondrial function decrease. These differences in the activation of NF-κB and Nrf2 that manifest in the prediabetic state and worsen through disease progression impart a heavy burden on the cardiovascular system, leading to increased oxidative damage. 

## 5. Conclusions

We highlight crosstalk between cardiac NF-κB and Nrf2 during progression of T2DM suggesting that impairment of Nrf2 is not only through lack of nuclear translocation but potentially through simultaneous activation of NF-κB. Most changes occur within 2 weeks of T2DM onset and are further exacerbated by 3 months and sustained by 6 months suggesting a permanent transition. Alterations in these key transcription factors illustrate the importance of mitigating chronic inflammation and bolstering antioxidant responses during early onset insulin resistance before it progresses to a frank T2DM condition.

## Figures and Tables

**Table 1:** Phenotype data for Sprague-Dawley (SD; *n* = 7), pre-diabetic UCD-T2DM (Pre; *n* = 9), 2-week diabetic UCD (2Wk; *n* = 9), 3-month diabetic UCD-T2DM (3Mo; *n* = 14), and 6-month diabetic UCD-T2DM (6Mo; *n* = 9) rats. * Significant difference from SD (*p* < 0.05). † Significant difference from Pre (*p* < 0.05). ‡ Significant difference from 2Wk (*p* < 0.05). § Significant difference from 3Mo (*p* < 0.05).

	SD	Pre	2Wk	3Mo	6Mo
Age (d)	168 ± 1	170 ± 3	168 ± 3	160 ± 2	265 ± 5
Body Mass (g)	404 ± 7	629 ± 5 *	645 ± 18 *	480 ± 13 *, †, ‡	474 ± 13 *, †, ‡
Fasting Glucose (mg/dL)	83 ± 1	96 ± 2 *	98 ± 5 *	189 ± 33 *, †, ‡	384 ± 30 *, †, ‡, §
Non-Fasting Glucose (mg/dL)	112 ± 2	146 ± 8 *	280 ± 30 *, †	508 ± 12 *, †, ‡	592 ± 4 *, †, ‡, §
HbA1c (%)	4.2 ± 0.2	4.1 ± 0.2	5.8 ± 0.4 *, †	12.0 ± 0.7 *, †, ‡	13.6 ± 0.7 *, †, ‡
Fasting Insulin (ng/mL)	0.6 ± 0.1	1.9 ± 0.2 *	2.4 ± 0.3 *	0.9 ± 0.1 †, ‡	0.5 ± 0.1 †, ‡, §

**Table 2:** Primers and GeneBank accession number for real-time PCR.

Primer Name	Nucleotide Sequence (5′-3′)	GeneBank Number
Keap1-F	ATGTGATGAACGGGGCAGTC	NM_057152.2
Keap1-R	AGAACTCCTCCTCCCCGAAG	
Gsk3β-F	CTGGCCACCATCCTTATCCC	NM_032080.1
Gsk3β-R	GAAGCGGCGTTATTGGTCTG	
Nrf2-F	ATTTGTAGATGACCATGAGTCGC	NM_031789.2
Nrf2-R	TGTCCTGCTGTATGCTGCTT	
Bach1-F	CACAAAGTGCAAAGACCCCG	NM_001107113.1
Bach1-R	ATCGCCTGACTGCTCGTATG	
Gclm-F	GTTCATTGTAGGATCG	NM_017305.2
Gclm-R	GGTGCCTATAGCAACAATCT	
Gclc-F	CTGGACTCATCCCCATTC	NM_012815.2
Gclc-R	GTAGTCAGGATGGTTTGC	
Hmox1-F	GAGCGAAACAAGCAGAACCC	NM_012580.2
Hmox1-R	ACCTCGTGGAGACGCTTTAC	
NF-κB-F	GAGCTGGTGGAGGCCCTG	NM_001276711.1
NF-κB-R	GACAGCGGCGTGGAGAC	
IKBα-F	CTCAAGAAGGAGCGGTTGGT	NM_001105720.2
IKBα-R	CCAAGTGCAGGAACGAGTCT	
Nox4-F	AGATGTTGGGCCTAGGATTGTG	NM_053524.1
Nox4-R	TGTGATCCGCGAAGGTAAGC	
B2m-F	ATGGGAAGCCCAACTTCCTC	NM_012512.2
B2m-R	ATACATCGGTCTCGGTGGGT	

## Data Availability

Data is contained within the article.
